# Computer Aided Classification of Neuroblastoma Histological Images Using Scale Invariant Feature Transform with Feature Encoding

**DOI:** 10.3390/diagnostics8030056

**Published:** 2018-08-28

**Authors:** Soheila Gheisari, Daniel R. Catchpoole, Amanda Charlton, Zsombor Melegh, Elise Gradhand, Paul J. Kennedy

**Affiliations:** 1 Centre for Artificial Intelligence, Faculty of Engineering and Information Technology, University of Technology Sydney, Ultimo, NSW 2007, Australia; soheila.gheisari@student.uts.edu.au; 2 The Tumour Bank, The Children’s Cancer Research Unit, The Kids Research Institute, The Children’s Hospital at Westmead, Locked Bag 4001, Westmead, NSW 2145, Australia; daniel.catchpoole@health.nsw.gov.au; 3 Department of Histopathology, Auckland City Hospital, Auckland 1023, New Zealand; acharlton@adhb.govt.nz; 4 Department of Molecular Medicine and Pathology, Faculty of Medical and Health Sciences, University of Auckland, Auckland 1142, New Zealand; 5 Department of Pathology, Southmead Hospital, Bristol BS10 5NB, UK; Zsombor.Melegh@nbt.nhs.uk; 6 Department of Cellular Pathology, Pathology Science Building, Southmead Hospital, Bristol BS10 5NB, UK; Elise.Gradhand@nbt.nhs.uk

**Keywords:** neuroblastoma, histological images, Scale Invariant Feature Transform (SIFT), feature encoding

## Abstract

Neuroblastoma is the most common extracranial solid malignancy in early childhood. Optimal management of neuroblastoma depends on many factors, including histopathological classification. Although histopathology study is considered the gold standard for classification of neuroblastoma histological images, computers can help to extract many more features some of which may not be recognizable by human eyes. This paper, proposes a combination of Scale Invariant Feature Transform with feature encoding algorithm to extract highly discriminative features. Then, distinctive image features are classified by Support Vector Machine classifier into five clinically relevant classes. The advantage of our model is extracting features which are more robust to scale variation compared to the Patched Completed Local Binary Pattern and Completed Local Binary Pattern methods. We gathered a database of 1043 histologic images of neuroblastic tumours classified into five subtypes. Our approach identified features that outperformed the state-of-the-art on both our neuroblastoma dataset and a benchmark breast cancer dataset. Our method shows promise for classification of neuroblastoma histological images.

## 1. Introduction

Neuroblastoma represents 8% of all malignancies in infants [1] and is the most common extracranial solid malignancy in childhood [2]. More than 15% of paediatric cancer deaths are the result of neuroblastoma [3]. Optimal management of neuroblastoma depends on many factors, one of which is the histopathological classification which is performed by pathologists using optical microscope to classify neuroblastic tumours of stained tissue sections. Pathologists use an optical microscope and classify neuroblastic tumours by examining thin slices of tissue on a glass slide. Pathologists commonly use the Shimada system [4] to classify neuroblastic tumours. Neuroblastic tumours are a heterogeneous group with complex features. A pathologist examines all the tissue with the microscope at low power magnification, and examines specific regions of the tissue at high power magnification. The classification of the neuroblastoma is based on the combination of these representative regions. However, it may be misleading for heterogeneous tumours [5]. Histopathologic classification is currently considered as the gold standard. Computer Aided Diagnosis (CAD) systems have the potential to augment and be complementary to human capability and may find new relationships which humans cannot detect [6]. Mohammad et al. [7] identified chronic lymphocytic leukemia using segmentation. They used the watershed technique to extract nucleus and cytoplasm mask. Sharma et al. [8] segmented nuclei in histological images based on intensity and morphological features. Zhang et al. [9] extracted features of breast cancer histological images using the combination of curvelet transform, Gray Level Co-occurrence Matrix (GLCM), and Completed Local Binary Pattern (CLBP) to classify them into cancer and non-cancer images. Spanhol et al. [10] extracted six different feature vectors from histological images of breast cancer in four magnification scales and classified them with four different classifiers. They used CLBP, LBP, Local Phase Quantization (LPQ), GLCM, Parameter-Free Threshold Adjacency Statistics (PFTAS), Oriented Fast and Rotated Brief (ORB) as the feature extractors. They classified the extracted features using 1-NN, Quadratic Discriminant Analysis (QDA), SVM and Random Forests (RF). They achieved accuracy ranges from 80% to 85%.

Moreover, CAD system is a promising tool to facilitate the classification of histological images of neuroblastic tumours with high throughput. So, there is an interest in developing approaches for computer assisted classification of neuroblastoma histological images. Research efforts on the classification of neuroblastoma histological images are divided into two groups: segmentation methods and feature extraction methods. Segmentation methods rely on morphological features such as size and shape of different cells. Kong et al. [11] classified neuroblastoma histological images by analyzing the whole slide images. They designed a multi-resolution approach to segment the histological images of neuroblastoma at each resolution level into cellular, neuropil and background. Finally, the system classified histological images of neuroblastoma into undifferentiated, poorly-differentiated, and differentiating tumour using k-nearest neighbor (k-NN) and Support Vector Machine (SVM) classifiers. Previous work from our group [12] applied a thresholding technique to neuroblastoma histological images to segment the regions of interest into undifferentiated and poorly-differentiated. However, inherent diversity of the appearance and size of the different cells in neuroblastoma histological images which belong to the same category makes their classification based on the segmentation more challenging.

Feature extraction methods on the other hand can extract diagnostic clues that might not be recognized by human eyes. A few feature extraction methods have been proposed for classification of neuroblastoma histological images which are based on the Local Binary Pattern (LBP) [13]. Sertel et al. [5] classified whole digitized slide haematoxylin and eosin (H&E) stained neuroblastoma tissue samples into stroma rich and stroma poor. The proposed algorithm is based on the extracted features using co-occurrence statistics and LBP. Previously, we [14] applied Patch Completed Local Binary Pattern (PCLBP) as a feature extractor on local patches and classified neuroblastoma histological images into five different classes using SVM classifier. All of the aforementioned feature extraction methods in classification of neuroblastoma histological images are based on the features which are not robust to scale variations.

**Figure 1:**
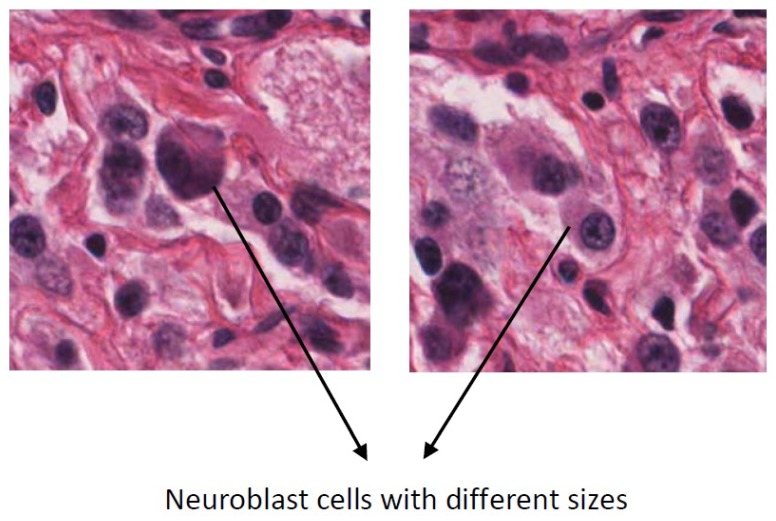
High intra-class variation in the size of the neuroblast cells.

We are unaware of any work specifically for neuroblastoma histological images that extract features which are robust to scale variations. In this study, we propose a CAD system based on Scale Invariant Feature Transform (SIFT) feature extraction method which is invariant to scale variations. This is useful for neuroblastoma histological images, in which size of the different cells in the same class has large variations. A sample of high intra-class variation in the size of the neuroblast cells is shown in Figure 1. We combine SIFT with the bag of features to refine more discriminative features for image classification. We have constructed a new dataset of neuroblastoma histological images to evaluate the proposed method.

The contributions of this paper are:

1. We applied SIFT to extract features of neuroblastoma histological images which are robust to scale variations.
2. We combine SIFT with the bag of features to reduce the number of features by extracting highly discriminative features.
3. We evaluate our method by comparing with other state-of-the-art benchmarks which show the effectiveness of our method in the classification of neuroblastoma histological images.
4. For more evaluation, we applied our method on a benchmark breast cancer dataset, BreaKHis dataset, and compared the results with state-of-the-art methods. Results show the effectiveness of our method.

The rest of this paper is as follows. Section 2 presents material and method. Section 3 shows the results. Section 4 presents the discussion, and finally Section 5 concludes the work.

## 2. Materials and Methods

### 2.1. Dataset Construction

**Figure 2:**
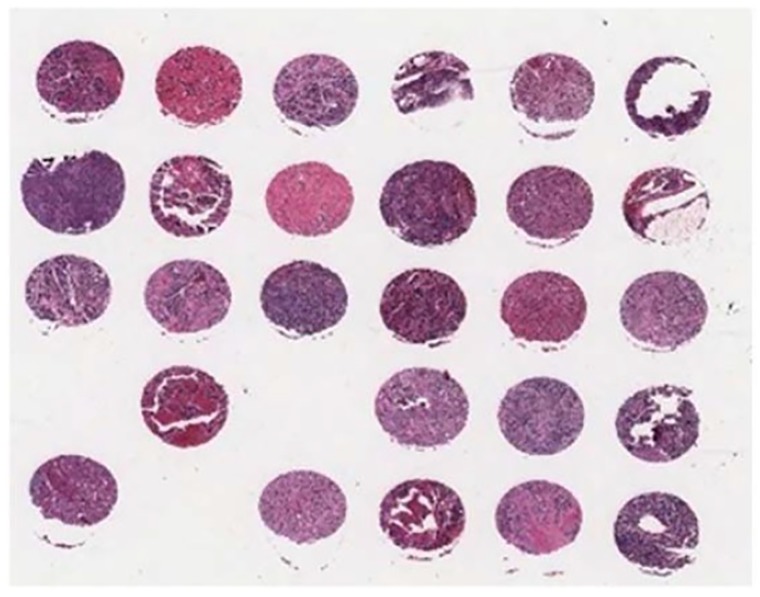
A sample of tissue microarray (TMA) slide.

**Figure 3:**
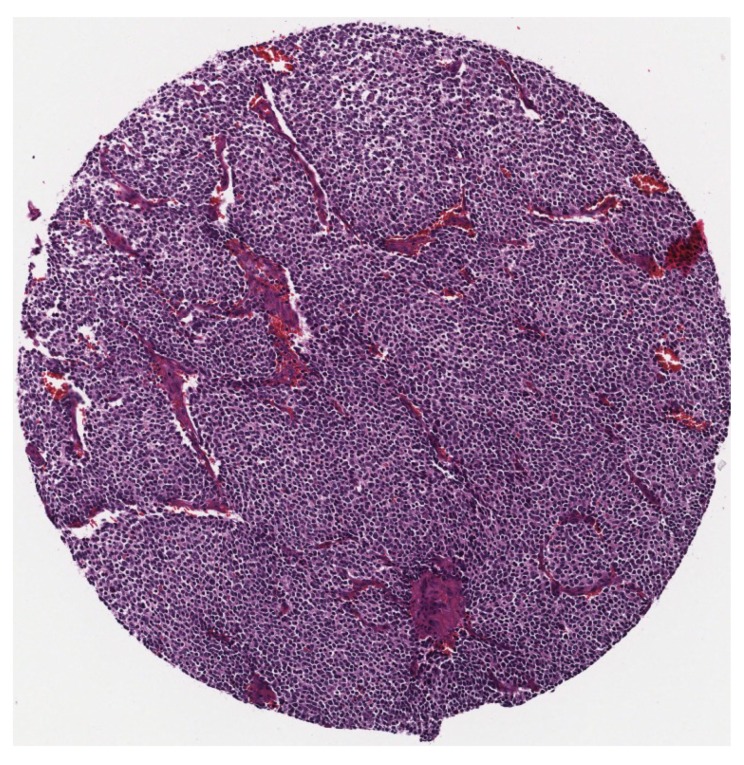
A sample of a single tumour.

As there is no public and available dataset in analysis of neuroblastic tumours we constructed a dataset which consists of images from neuroblastic tumours from The Tumour Bank of the Kid’s Research Institute at The Children’s Hospital at Westmead, Sydney, Australia. All the specific details of patients were removed from the dataset and a de-identified dataset was used in this research. The initial dataset was generated from cancer tissue biopsy slides and consisted of images of H&E stained tissue microarrays (TMA) of neuroblastic tumours. We used six TMA slides and seven whole sections representing 125 patients. Each TMA slide contains from 20 to 40 cores of neuroblastic tumour. Samples of TMA slide containing cores of different neuroblastic tumours are shown in Figure 2 and Figure 3, respectively. The diameter of TMA cores is 1.2 mm, stained with H&E and cut at 3 μm thickness. Although most images belong to different patients, some of them are duplicates. Our constructed dataset is much larger in terms of patients and images than the datasets used by Tafavogh et al. [12] and Kong et al. [11].

**Figure 4:**
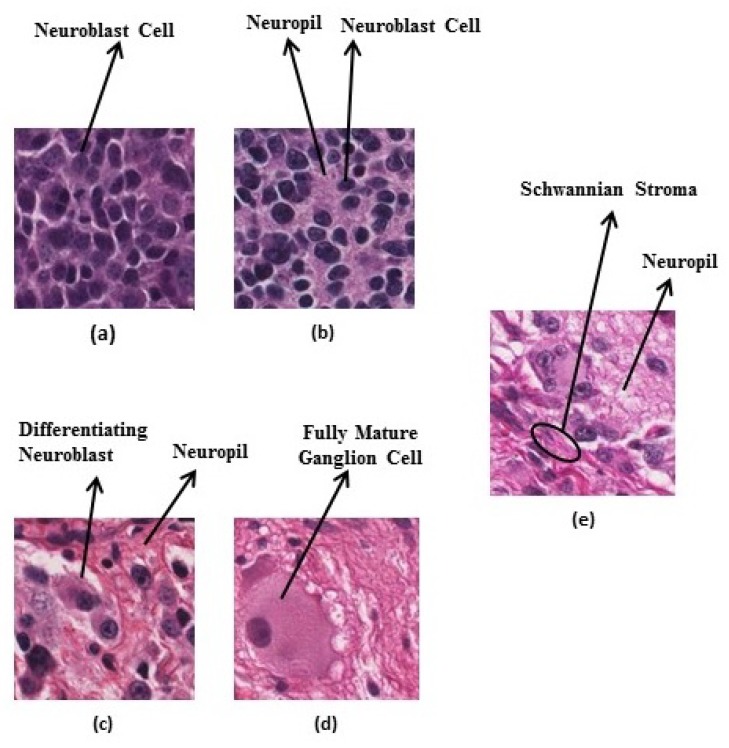
Neuroblastic tumour categories: (**a**) undifferentiated neuroblastoma, (**b**) poorly-differentiated neuroblastoma, (**c**) differentiating neuroblastoma, (**d**) ganglioneuroma, and (**e**) ganglioneuroblastoma.

All collected tissue samples were classified as undifferentiated neuroblastoma, poorly-differentiated neuroblastoma, differentiating neuroblastoma, ganglioneuroblastoma, or ganglioneuroma by pathologists. Representative images in the categories are shown in Figure 4.

Due to the size of the scanned neuroblastic tumour images, up to a gigabyte each, the computational cost is high. Therefore, they were cropped to equal-sized square regions (300 × 300 pixels) by an expert histopathologist [AC] in which all the specification of a particular subtype of neuroblastic tumour is present in the cropped image. This is the best size which is large enough to capture diagnostic features of each category and small enough for the computational cost. Table 1 shows the number of images in our dataset.

### 2.2. Method

**Figure 5:**
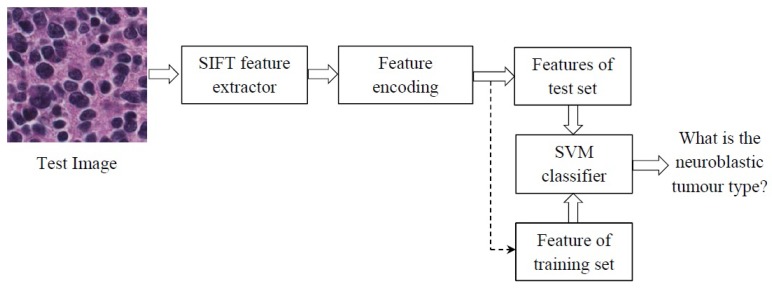
The scheme of the proposed method. SIFT, Scale Invariant Feature Transform; SVM, Support Vector Machine.

The overall framework of our approach is shown in Figure 5. The Scale Invariant Feature Transform (SIFT) extracts features from the test images and feeds them into the feature encoding block to summarize them into new discriminative features. Here, a SVM is used to classify the test image by comparing their extracted features with the training set images.

#### 2.2.1. Scale Invariant Feature Transform

The SIFT was proposed by Lowe in 2004 [15]. In this method, distinctive image features are extracted from the scale-invariant keypoints. SIFT features are invariant to rotation and robust to scale and illumination variations. SIFT algorithm consists of four stages: detection of scale-space extrema, keypoint localization, orientation assignment and descriptor representation. These are described in detail below.

#### Detection of Scale-Space Extrema

The first step involves searching over multiple scales and image locations to identify keypoints that are invariant to differences in scale. Detecting the locations that are invariant to scale changes in an image is determined using a scale-space function [16]. Lindeberg [17] showed that the only possible scale-space kernel is the Gaussian function, G(x,y). Scale space of an image, L(x,y,σ), is calculated by the convolution of a variable-scale Gaussian function, G(x,y,σ), with an input image, I(x,y).

L(x,y,σ)=G(x,y,σ)×I(x,y)

and

$$G\left(x,y,\sigma \right)=\frac{1}{2\pi \sigma^{2}}e^{-\left(x^{2}+y^{2}\right)/2\sigma^{2}}$$

where *x* and *y* are image coordinates and σ is width of the Gaussian function. Stable keypoint locations are defined by Difference of Gaussian (DoG) function in the scale-space. The scale-space extrema is located in *D*(*x*,*y*,σ), which is calculated as the difference between two images, one with scale *k* and the other one with scale kσ, k is a multiplicative factor.

D(x,y,σ)=(G(x,y,kσ)−G(x,y,σ))×I(x,y)=L(x,y,kσ)−L(x,y,σ)

**Figure 6:**
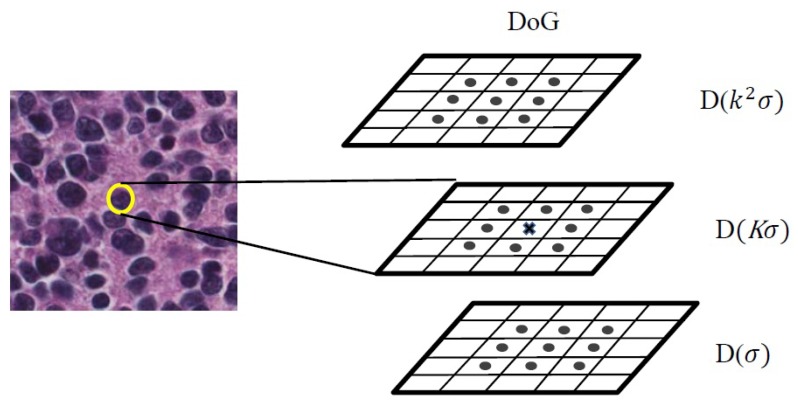
The scheme of keypoint detecting. DoG, Difference of Gaussian.

Points in the DoG function which are maxima or minima among the 26-neighboring pixels (eight neighbors in the current image and nine neighbors in the above and nine neighbors in the below scales) are considered as extrema points which are called keypoints. The detection of keypoints is shown in Figure 6.

#### Keypoint Localization

Some of the extracted keypoints that have low contrast or are poorly localized on edges of the image are not stable. These keypoints are eliminated in this stage. Location of the keypoint, *z*, is achieved by calculating the Laplacian.

$$z=-\frac{\partial^{2}D^{-1}}{\partial x^{2}}\frac{\partial D}{\partial x}$$

If the value of Laplacian function is less than a threshold value, called the contrast threshold ($C_{C}$), the point is rejected. Therefore, the extracted keypoints with low contrast are eliminated. Some of the extracted keypoints have a strong response along edges. Therefore, we need to eliminate these points to increase the stability. Lowe et al. [15] used a 2×2 Hessian matrix, a square matrix of second-order partial derivatives of a scalar-valued function which describes the local curvature of a function of variables, to compute two eigenvalues in edges. If the ratio between the larger and smaller eigenvalue is greater than the edge threshold ($C_{E}$), that keypoint is discarded. Therefore, we have more stable keypoints after eliminating the low contrast and edge keypoints. The values of $C_{C}$ and $C_{E}$ are set in the experimental setup subsection.

#### Orientation Assignment

Before extraction of a descriptor for the keypoint, the keypoint is assigned an orientation to make it invariant to rotation. The orientation of the keypoint is calculated from the orientation histogram of local gradients from the closest smoothed image L(x,y,σ) which means there is a potential keypoint at (x,y) with scale σ. The gradient magnitude and the orientation are calculated as follow.

$$m\left(x,y\right)=\sqrt{{\left(L(x+1,y)-L(x-1,y)\right)}^{2}+(L\left(x,y+1\right)-L\left(x,y-1\right){)}^{2}}$$

$$\theta \left(x,y\right)=\tan^{-1}\frac{L(x,y+1)-L(x,y-1)}{L(x+1,y)-L(x-1,y)}$$

**Figure 7:**
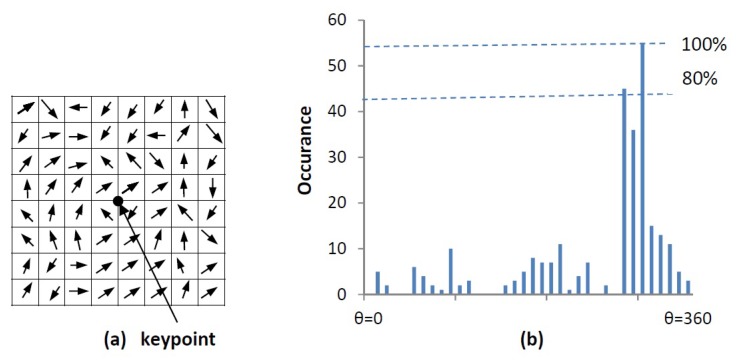
Orientation calculation: (**a**) gradient orientations of sample points within a region around the keypoint (**b**) orientation histogram.

An orientation histogram is created from the gradient orientations of sample points within a region around the keypoint. It consists of 36 bins covering the 360 degrees of orientation [15]. Each sample added to the histogram is weighted by the gradient magnitude, m(x,y), and by a circular gaussian with σ equal to 1.5 times the scale of the keypoint [15]. Any peaks above 80% of the maximum peak height are considered to calculate the orientation, it creates keypoints with same location and scale, but different directions. An accurate orientation is calculated using the interpolation of dominant peaks in the histogram. The orientation calculation is illustrated in Figure 7.

#### Descriptor Representation

**Figure 8:**
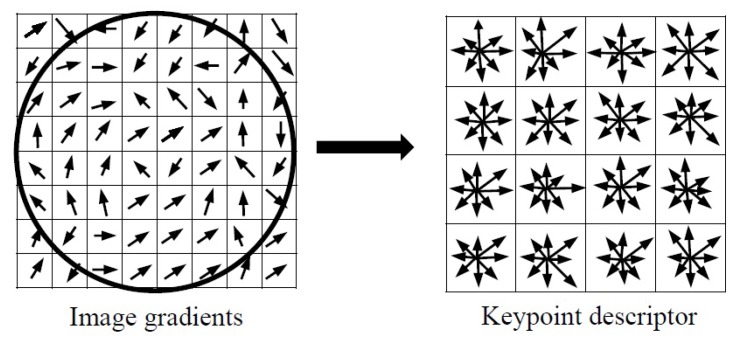
Keypoint descriptor: (**left**) gradient magnitude and orientation are calculated in a region around the keypoint (**right**) a 16×16 neighborhood around the keypoint is divided to 4×4 subregions. In each subregion the gradient vectors are accumulated into 8-bins orientation histograms.

In this stage, a SIFT descriptor is calculated for each keypoint as a histogram of orientation in eight directions (as shown in Figure 8). According to [15,18,19], it is common that a 16 × 16 neighborhood region around each keypoint is divided into 4 × 4 sub-regions. The gradient vectors are aggregated into 8-bin histogram over the 4×4 matrix of subregions. Then, SIFT extracts 4×4×8=128 element feature vector for each keypoint.

SIFT has three free parameters which must be set. The first one is σ, the width of the Gaussian. Increasing σ reduces the number of features generated from an image [20]. The second free parameter is the contrast threshold ($C_{C}$) which eliminates keypoints with low contrast. The third free parameter is the edge threshold ($C_{E}$) which eliminates the unstable keypoints that are near the edges. These parameters are set in Section 5.

#### 2.2.2. Feature Encoding

**Figure 9:**
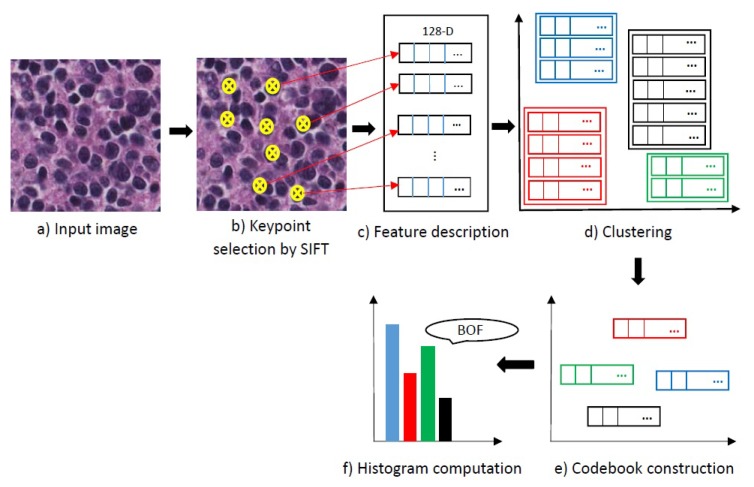
Scheme of the feature encoding block. Different colours indicate different clusters and different codewords. BOF, Bag of Features.

The SIFT-descriptor extracts a feature vector with 128 elements for each keypoint. Using all of the extracted features in the classification of neuroblastoma histological images is time-consuming and have a high computational complexity. So, we use a bag of features [21] as a feature encoding to compute more discriminative representations. The scheme of the bag of features is shown in Figure 9. It starts by selecting keypoints, step (b), and then describes keypoints from the input image step (c). In the next step (d), the extracted features are clustered and the codebook which consists of the codewords is constructed in step (e), codewords are the extracted features by SIFT. Then, a bag of features histogram counts the occurrences of each feature in step (f).

We use clustering to construct a codebook. All the extracted keypoints are clustered to find a set of centroids. In this work, we use k-means algorithm [22] for clustering. An important factor in construction of the codebook is selection of the number of codewords. We evaluate different codebook sizes and select the best one; experimental results are shown in Section 5. The input image is represented by a histogram of codewords using the feature encoding block. In the final stage, histological images are classified by training a classifier which classifies the histograms of the feature encoding block. We use SVM classifier which can efficiently perform a non-linear classification using an optimal hyperplane in a high-dimensional space.

## 3. Results

In this section, we evaluate the performance of the proposed approach on the constructed dataset of histological images of neuroblastoma. The database is divided randomly into three subsets: the first part for training (623 images), the second part for validation (211 images) and the third part for testing (209 images). We use the validation set to select the optimum values for free parameters of the proposed approach. Finally, the system is evaluated using testing and training sets.

### 3.1. Experimental Setup

SIFT has three parameters to be selected: Gaussian σ, contrast threshold ($C_{C}$), and edge threshold ($C_{E}$). We randomly divide the validation set into two subsets including 150 and 61 images. We train the algorithm using 150 images and compute the accuracy of the algorithm using 61 images to tune the free parameters of the algorithm. We repeat the above procedure multiple times (10 times) and compute the average over all experiments to have an accurate result. We tested SIFT with σ = 0.1, as $C_{C}$ and $C_{E}$ are 0.02 and 5, respectively. We increased the σ by step size 0.4 and found the best accuracy for the proposed system. According to Table 2, the best accuracy found when σ was 1.7. So, we consider σ 1.7 in the next experiments.

We completed another experiment to find the optimum value for $C_{C}$ while the $C_{E}$ is 5. According to Table 3, the best accuracy was found when the $C_{C}$ is 0.04. Also, we chose the optimum value for $C_{E}$. We tested five different values for $C_{E}$ and according to the Table 4 found the best accuracy when $C_{E}$ is 11. Furthermore, we tested the proposed system for five different codebook sizes, starting with 300 and following with 400, 500, 600 and 700. Table 5 shows the average classification accuracy versus size of the codebook. The best accuracy was found when the codebook size was 500. This value was set for the next experiments.

For SVM, we tried different kernels: linear, polynomial, Radial Basis Function (RBF), and sigmoid. We retained the RBF kernel which produced the best results. The kernel parameter γ was empirically defined through experiments with the best value of 0.004.

### 3.2. System Evaluation

**Figure 10:**
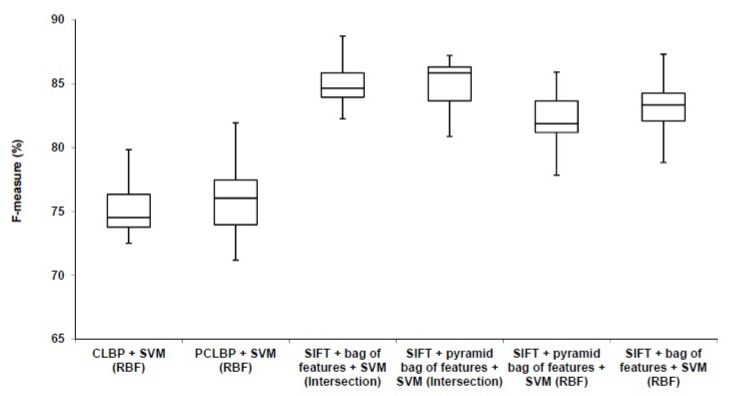
Comparison between our algorithm (SIFT + bag of feature) with the benchmarks (CLBP) and (PCLBP).

To evaluate the proposed method, we use the remaining 80% of the dataset which is not seen in the experimental setup. They are divided randomly into the training set (623 images) and testing set (209 images). We train the final model using the training set and test it using the testing set. We repeat this procedure multiple times (10 times) and report the average accuracy. We use the standard bag of features and the pyramid bag of features [21] algorithms to evaluate the proposed method. In each case, we used RBF kernel type and histogram intersection kernel [23]. We measured the performance of our algorithm by the weighted average (weighted by the number of samples in each class) F-measure, recall and precision. The results are shown in Table 6. We compared our method with Patched Completed Local Binary Pattern (PCLBP) and Completed Local Binary Pattern (CLBP) as benchmarks. Distribution of the computed F-measures for different approaches over the ten trials is presented in Figure 10. It shows that combination of SIFT with the bag of feature works better than CLBP and PCLBP.

We completed a *t*-test (with significance level of 5%) giving *p*-values of $15\times 10^{-7}$ and $12\times 10^{-5}$ for CLBP and PCLBP with histogram intersection kernel SVM, respectively. The results showed that the combination of SIFT with bag of features and histogram intersection kernel SVM statistically significantly improved the classification accuracy in comparison with CLBP and PCLBP with RBF kernel SVM. The F-measure accuracy of our proposed method in the best case is 10.04% and 9.23% higher than CLBP and PCLBP, respectively. The weighted average of precision, recall, and F-measure of our algorithm is better than PCLBP and CLBP. Table 7 shows the representative confusion matrix. Interestingly, the poorest computer performance was in discriminating between poorly-differentiated and differentiating neuroblastoma, a distinction that human pathologists also find difficult in limited fields of view.

### 3.3. Comparison to Other Datasets

We tested our algorithm on the collected dataset which consisted of 1043 (300 × 300 pixels) neuroblastoma histological images. As there was no public and available dataset in this field, we evaluated the proposed algorithm on a second dataset provided by the University of Bristol. The dataset consists of five whole tissue sections, one ganglioneuroblastoma, three poorly-differentiated neuroblastoma and one ganglioneuroma. To evaluate the proposed method, we use 623 sub-images cropped from Children’s Hospital at Westmead dataset as training set and five whole tissue sections from the university of Bristol as the testing set. Here, we randomly select ten sub-images (300 × 300 pixels) from each whole tissue section. First, the algorithm assigns a label to each sub-images. Then, each tissue section is classified using the majority vote among ten labels corresponding to ten sub-images. The whole tissue section predicted labels and result of majority vote are shown in Table 8.

**Figure 11:**
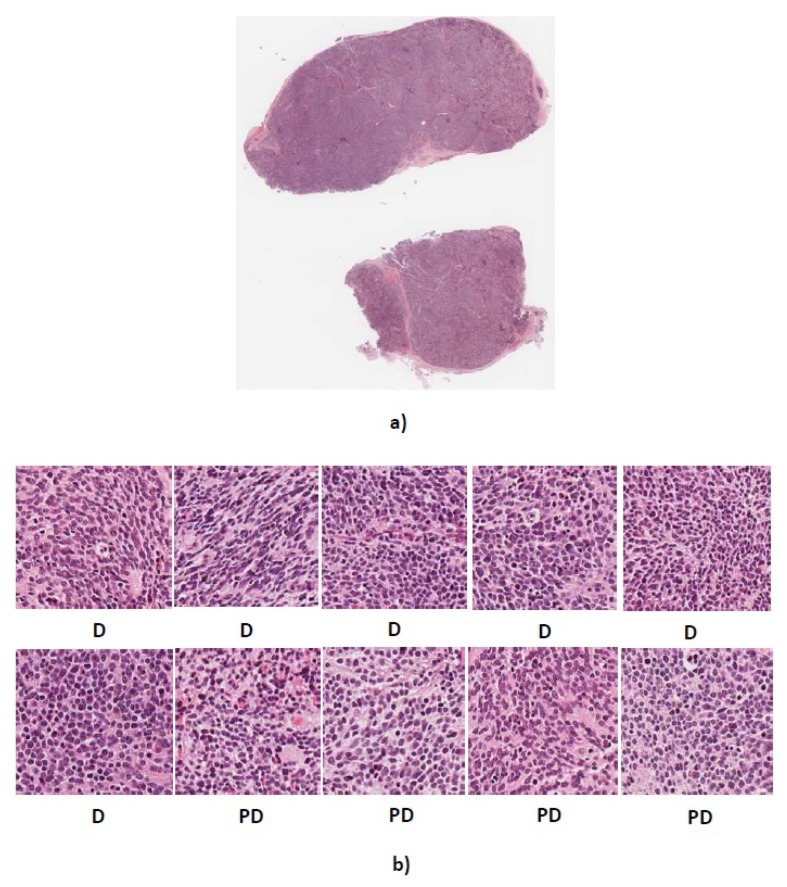
(**a**) Whole tissue section 4906 with actual label PD (**b**) Predicted labels for ten randomly cropped images from whole tissue section. D, differentiating neuroblastoma; PD, poorly-differentiated neuroblastoma.

**Figure 12:**
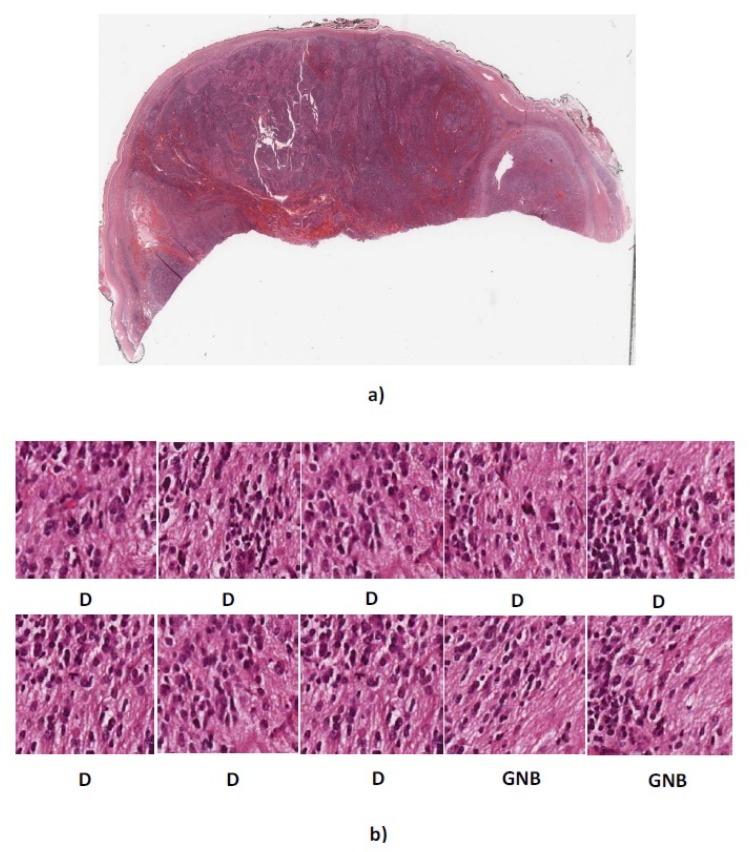
(**a**) Whole tissue section 4909 with actual label GNB (**b**) Predicted labels for ten randomly cropped images from whole tissue section. D, differentiating neuroblastoma; GNB = ganglioneuroblastoma.

As can be seen, our algorithm correctly classified three out of five whole tissue sections and the other two were misclassified as differentiating type while their actual types are poorly-differentiated and ganglioneuroblastoma. Figure 11 and Figure 12 show the sub-images for whole tissue section 4906 and 4909.

The actual label of whole tissue section 4906 is poorly-differentiated. The four out of ten sub-images extracted from 4906 were predicted as poorly-differentiated neuroblastoma and the other six images were predicted as differentiating neuroblastoma. The proposed algorithm assigns label differentiating to the whole tissue section 4906 based on the majority vote. Moreover, two out of ten extracted sub-images from 4909 with actual label ganglioneuroblastoma were predicted as ganglioneuroblastoma and the others predicted as differentiating. Therefore, according to the majority vote, the whole tissue section 4909 was predicted as differentiating.

For more evaluation, we applied the proposed algorithm on the BreaKHis breast cancer dataset [10]. It consists of 7909 breast cancer histopathology images divided into benign and malignant tumours. The images are in five different levels of magnification with dimension of 700 × 460 pixels. The image distribution is shown in Table 9.

Spanhol et al. [10] applied combination of six feature extractors (i.e., CLBP, LBP, Gray Level Co-occurrence Matrices (GLCM), Local Phase Quantization (LPQ), Oriented Fast Rotated Brief (ORB) and Parameter-Free Threshold Adjacency Statistics (PFTAS)) with 1-Nearest Neighbor (1-NN), Quadratic Discriminant Analysis (QDA), Random Forest (RF) and SVM classifiers to classify breast cancer histopathology images. They calculated average recognition rates of five trials for magnification factors of 40×, 100×, 200× and 400×. They achieved the best average recognition rates using the PFTAS as a descriptor. Table 10 shows the best average recognition rates of the classifiers trained with PFTAS descriptor.

For comparison, we follow the same testing protocol in their paper. The BreaKHis dataset is randomly divided into a training set (70%) and a testing set (30%). We train the model using the training set and test using the testing set and compute the recognition rates. We repeat this procedure five times and report the average recognition rates for all magnification factors. The results are shown in Table 11. According to Spanhol et al. [10] the recognition rate is calculated as follow

$$Recognition\;Rate=\frac{\sum Patient\;Score}{Total\;number\;of\;patients}$$

$$Patient\;Score=\frac{N_{rec}}{N_{P}}$$

where $N_{rec}$ is the number of images for each patient which are correctly classified and $N_{P}$ is the number of images for patient P existing in the dataset. The results are shown in Table 11. Results show that the combination of SIFT with bag of features works better than the methods that Spanhol et al. [10] tested.

## 4. Discussion

We used SIFT as a feature extractor and combined it with bag of features. It is a new approach to classify neuroblastoma histological images into five groups. Although several methods have been proposed in the literature, our system has multiple advantages over those systems:

1. It is the first time that neuroblastoma histological images with a complex texture are classified into five classes using features that are robust to scale variations.
2. We apply the bag of features algorithm to refine the extracted features by SIFT and improve the classification accuracy.
3. The poorest performance of our algorithm is in discriminating between poorly differentiated and differentiating neuroblastoma, a distinction that human pathologists also find difficult in limited fields of view. This is the reflection of why our model does same as the pathologists.
4. As there is no public and available dataset of neuroblastoma histological images, we have evaluated our algorithm under the BreaKHis dataset which is a public and available breast cancer dataset. Moreover, we evaluated our algorithm on the second dataset of neuroblastoma tumour provided by the University of Bristol.
5. Pathologists scan slides, naturally pick out local regions and decide about type of the tumour based on these regions. Our experimental design sought to emulate that process through evaluation of cropped images. Ten sub-images are extracted randomly from each whole tissue section which was provided by the University of Bristol. Then, the whole tissue section is classified using the majority vote among the ten labels assigned to the ten sub-images. As shown in Figure 11 and Figure 12, the identification of visual features that distinguish our cropped images is not immediately apparent. However, the extracted features by SIFT are the vectors with 128 elements, they are mathematical features not visual features. It still remains to be determined whether these mathematical features represent biological distinctions within the tumours that have a clinical sequelae.
6. Our ‘gold standard’ is the human eye and pathologist assessment. But we also know that the human eye is gilded by subjectivity and that if we had multiple pathologist look over the cropped images our specificity would likely not be 100%. For example distinction between poorly-differentiated and differentiating tumour types is known to be difficult for pathologists to distinguish, which is reflected within our analysis (Table 7). That said, SIFT has not given us 100% specificity either. Perhaps that is a reflection of fidelity of the algorithm to find really minor variations, possibly unobservable to the naked eye. Perhaps it is that SIFT is not as good as the naked eye? This distinction has not been tested in this paper and is beyond the scope of the immediate study. However, it does highlight that SIFT may have the potential to delve deeper into the images of neuroblastoma tumours and identify new characteristics that relate to tumour growth and treatment response. As the future direction of this study, we would give 1043 cropped images to be classified by completely different pathologists and see the classification differences between them and would compare to the results from the SIFT algorithm.
7. Our experimental results show that combination of SIFT with the bag of features is a promising tool for classification of neuroblastoma histological images but at this stage the specificity of SIFT has not been shown to improve on the specificity of visual analysis by a pathologist. Consequently, further testing of human-computer differences needs to be undertaken.

## 5. Conclusions

We proposed combination of Scale Invariant Feature Transform (SIFT) and bag of features to classify histological images of neuroblastic tumours into five categories using the SVM classifier. The algorithm built the feature vectors by extraction of 128 element vectors. The advantage of the proposed method is the extraction of features which are robust to scale variations and combine them with the bag of features to yield more discriminative features which increased the classification accuracy. We evaluated the proposed algorithm on the constructed dataset with 1043 cropped images of neuroblastoma from samples of five categories. As there is no public and available dataset of neuroblastoma histological images, we tested our proposed method under the BreaKHis breast cancer dataset for more evaluation. Results indicate that a CAD system based on combination of SIFT with bag of features outperforms the state-of-the-art methods on both neuroblastoma and breast cancer datasets.

## Figures and Tables

**Table 1:** Number of different categories of neuroblastic tumour cropped images.

Category of Neuroblastic Tumour	Number of Cropped Images	Number of Patients
poorly-differentiated	571	77
differentiating	187	12
undifferentiated	155	10
ganglioneuroma	84	18
ganglioneuroblastoma	46	8
**Total**	**1043**	**125**

**Table 2:** Average classification accuracy of the SIFT over neuroblastic tumour dataset using different values for σ. Bold value indicates the highest classification accuracy.

σ	Classification Accuracy (%)
0.1	73.02
0.5	73.25
0.9	75.24
1.3	76.52
1.7	**78.96**
2.1	76.24

**Table 3:** Average classification accuracy of the SIFT over neuroblastic tumour dataset using different values for contrast threshold ($C_{C}$). Bold value indicates the highest classification accuracy.

Contrast Threshold	Classification Accuracy (%)
0.02	73.19
0.03	76.21
0.04	**76.58**
0.05	75.68
0.06	74.25

**Table 4:** Average classification accuracy of the SIFT over neuroblastic tumour dataset using different values for edge threshold ($C_{E}$). Bold value indicates the highest classification accuracy.

Edge Threshold	Classification Accuracy (%)
5	78.25
11	**81.76**
17	77.24
23	76.15
29	74.18

**Table 5:** Average classification accuracy of the SIFT over neuroblastic tumour dataset using different codebook sizes. Bold value indicates the highest classification accuracy.

Size of the Codebook	Classification Accuracy (%)
300	78.2
400	80.31
500	**82.25**
600	79.77
700	81.62

**Table 6:** Weighted average precision, recall and F-measure of the proposed method and benchmarks. Bold value indicates the highest F-measure. CLBP, Completed Local Binary Pattern; SVM, Support Vector Machine; RBF, Radial Basis Function; PCLBP, Patched Completed Local Binary Pattern; SIFT, Scale Invariant Feature Transform.

Method	Precision (%)	Recall (%)	F-measure (%)
CLBP + SVM (RBF)	74.1 ± 2.35	76.25 ± 2.23	75.15 ± 2.28
PCLBP + SVM (RBF)	75.59 ± 3.15	76.35 ± 3.41	75.96 ± 3.27
SIFT + bag of features + SVM (RBF)	81.62 ± 3.72	84.54 ± 1.66	83.03 ± 2.63
SIFT + pyramid bag of features + SVM (RBF)	80.63 ± 3.55	83.57 ± 1.81	82.08 ± 2.65
SIFT + bag of features + SVM (histogram intersection)	83.81 ± 3.33	86.61 ± 1.87	**85.19 ± 2.42**
SIFT + pyramid bag of features + SVM (histogram intersection)	83.5 ± 3.64	86.41 ± 1.22	84.93 ± 2.35

**Table 7:** A representative confusion matrix for dataset from Children’s Hospital at Westmead.

				Predicted		
		Differentiating	Ganglio-	Ganglio-	Poorly-	
					differentiated	Undifferentiated
		neuroblastoma	neuroma	neuroblastoma	neuroblastoma	neuroblastoma
	Differentiating					
	neuroblastoma	6	1	4	12	0
	Ganglioneuroma	0	14	5	0	0
**Actual**	Ganglio-					
	neuroblastoma	1	0	34	0	0
	Poorly-					
	differentiated					
	neuroblastoma	8	1	0	119	0
	Undifferentiated					
	neuroblastoma	0	0	0	2	1

**Table 8:** The actual and predicted labels for dataset from University of Bristol.

Tissue Section Number	Actual Label	Predicted Label	Majority Vote
4905	ganglioneuroma	ganglioneuroma	10 out of 10
4906	poorly-differentiated	differentiating	4 out of 10
4907	poorly-differentiated	poorly-differentiated	8 out of 10
4908	poorly-differentiated	poorly-differentiated	9 out of 10
4909	ganglioneuroblastoma	differentiating	2 out of 10

**Table 9:** Image distribution of BreaKHis dataset by magnification factor and class.

Magnification	Benign	Malignant	Total
40×	625	1370	1995
100×	644	1437	2081
200×	623	1390	2013
400×	588	1232	1820
**Total**	2480	5429	7909
**No of Patients**	24	58	82

**Table 10:** Best average recognition rates (%) of the classifiers trained with different descriptors tested by Spanhol et al. [10] on BreaKHis dataset. Bold values indicate the highest recognition rate in each magnification. PFTAS, Parameter-Free Threshold Adjacency Statistics; 1-NN, 1-Nearest-Neighbor; QDA, Quadratic Discriminant Analysis; RF, Random Forest; SVM, Support Vector Machine.

Descriptor	Classifier	40×	100×	200×	400×
PFTAS	1-NN	80.9 ± 2.0	80.7 ± 2.4	81.5 ± 2.7	79.4 ± 3.9
PFTAS	QDA	**83.8 ± 4.1**	**82.1 ± 4.9**	84.2 ± 4.1	78.2 ± 5.9
PFTAS	RF	81.8 ± 2	81.3 ± 2.8	83.5 ± 2.3	81.0 ± 3.8
PFTAS	SVM	81.6 ± 3.0	79.9 ± 5.4	**85.1 ± 3.1**	**82.3 ± 3.8**

**Table 11:** Average recognition rate (%) of our proposed method and Spanhol’s method in different magnifications. Bold values indicate the highest average recognition rate in different magnifications. SIFT, Scale Invariant Feature Transform; SVM, Support Vector Machine; RBF, Radial Basis Function.

Method	40×	100×	200×	400×
Spanhol et al. [10]	83.8 ± 4.1	82.1 ± 4.9	85.1 ± 3.1	82.3 ± 3.8
SIFT + bag of features + SVM (RBF)	82.86 ± 1.51	85.11 ± 0.87	81.55 ± 0.78	84.15 ± 0.69
SIFT + pyramid bag of features + SVM (RBF)	80.07 ± 1.56	80.75 ± 3.91	76.31 ± 0.99	79.21 ± 2.85
SIFT + bag of features + SVM (histogram intersection)	**85.4 ± 1.39**	**87.25 ± 2.51**	**85.6 ± 0.76**	**86.04 ± 1.56**
SIFT + pyramid bag of features + SVM (histogram intersection)	83.06 ± 1.4	85.6 ± 0.43	81.29 ± 1.64	84.65 ± 0.25

## References

[1] Ries L., Smith M.A., Gurney J.G., Linet M., Tamra T., Bunin J.L.Y.G.R., Linet M., Tamra T., Young J.L., Bunin G.R. (1999). Cancer Incidence and Survival among Children and Adolescents.

[2] Brodeur G.M., Maris J.M. (1993). Principles and Practice of Pediatric Oncology.

[3] Park J., Caron H., Eggert A. (2008). Neuroblastoma: Biology, Prognosis, and Treatment. Paediatric Clin. N. Am..

[4] Shimada H., Ambros I., Dehner L., Hata J., Joshi V., Roald B., Stram D., Gerbing R., Lukens J., Matthay K., Castleberry R. (1999). The International Neuroblastoma Pathology Classification (the Shimada System). Cancer.

[5] Sertela O., Kong J., Shimadac H., Catalyurek U., Saltz J., Gurcan M. (2009). Computer-aided Prognosis of Neuroblastoma on Whole-slide Images: Classification of Stromal Development. J. Patt. Recognit..

[6] Yu K., Zhang C., Berry G., Altman R., Re C., Rubin D., Snyder M. (2016). Predicting Non-Small Cell Lung Cancer Prognosis by Fully Automated Microscopic Pathology Image Features. Nat. Commun..

[7] Mohammed E., Mohamed M., Naugler C., Far B. Chronic Lymphocytic Leukemia Cell Segmentation from Microscopic Blood Images using Watershed Algorithm and Optimal Thresholding. Proceedings of the 26th IEEE Canadian Conference on Electrical and Computer Engineering.

[8] Sharma H., Zerbe N., Heim D., Wienert S., Behrens H., Hellwich O., Hufnagl P. A Multi- Resolution Approach for Combining Visual Information using Nuclei Segmentation and Classification in Histopathological Images. Proceedings of the 10th International Conference on Computer Vision Theory and Applications.

[9] Zhang Y., Zhang B., Lu W. (2013). Breast Cancer Histological Image Classification with Multiple Features and Random Subspace Classifier Ensemble. Stud. Comput. Intell..

[10] Spanhol F., Oliveira L., Caroline P., Laurent H. (2016). A Dataset for Breast Cancer Histopathological Image Classification. IEEE Trans. Biomed. Eng..

[11] Kong J., Sertel O., Shimada H., Boyer K., Saltz J., Gurcan M. (2009). Computer-Aided Evaluation of Neuroblastoma on Whole-Slide Histology Images: Classifying Grade of Neuroblastic Differentiation. Pattern Recognit..

[12] Tafavogh S., Meng Q., Catchpoole D.R., Kennedy P.J. Automated Quantitative and @ualitative Analysis of the Whole Slide Images of Neuroblastoma Tumour for making a prognosis decision. Proceedings of the IASTED 11th International Conference on Biomedical Engineering.

[13] Ojala T., Pietikainen M., Maenpaa T. (2002). Multiresolution Gray-Scale and Rotation Invariant Texture Classification with Local Binary Patterns. IEEE Trans. Pattern Anal. Mach. Intell..

[14] Gheisari S., Catchpoole D.R., Charlton A., Kennedy P.J. Patched Completed Local Binary Pattern is an Effective Method for Neuroblastoma Histological Image Classification. Proceedings of the 15th Australian Data Mining Conference.

[15] Lowe D.G. (2004). Distinctive Image Features from Scale-Invariant Keypoints. Int. J. Comput. Vis..

[16] Witkin A. Scale-space Filtering. Proceedings of the International Joint Conference on Artificial Intelligence.

[17] Lindeberg T. (1993). Detecting salient blob-like image structures and their scales with a scale-space primal sketch: A method for focus-of-attention. Int. J. Comput. Vis..

[18] Smith D., Harvey R. (2011). Document Retrieval Using SIFT Image Features. J. Univ. Comput. Sci..

[19] Nam J.E.J., Maurer M., Mueller K. (2009). A High-dimensional Feature Clustering Approach to Support Knowledge-assisted Visualization. Comput. Graph..

[20] Tang C.Y., Wu Y.L., Hor M.K., Wang W.H. Modified SIFT Descriptor for Image Matching Under Interference. Proceedings of the International Conference on Machine Learning and Cybernetics.

[21] Lazebnik S., Schmid C., Ponce J. Beyond Bags of Features: Spatial Pyramid Matching for Recognizing Natural Scene Categories. Proceedings of the Computer Vision and Pattern Recognition (CVPR).

[22] Kanungo T., Mount D.M., Netanyahu N.S., Piatko C.D., Silverman R., Wu A.Y. (2002). An Efficient k-Means Clustering Algorithm: Analysis and Implementation. IEEE Trans. Pattern Anal. Mach. Intell..

[23] Maji S., Berg A.C., Malik J. Classification Using Intersection Kernel Support Vector Machines is Efficient. Proceedings of the Computer Vision and Pattern Recognition (CVPR).

